# Does noise affect learning? A short review on noise effects on cognitive performance in children

**DOI:** 10.3389/fpsyg.2013.00578

**Published:** 2013-08-30

**Authors:** Maria Klatte, Kirstin Bergström, Thomas Lachmann

**Affiliations:** Center for Cognitive Science, Cognitive and Developmental Psychology Laboratory, University of KaiserslauternKaiserslautern, Germany

**Keywords:** noise, cognitive performance, cognitive development, children, speech perception, listening comprehension, irrelevant sound effect, classroom acoustics

## Abstract

The present paper provides an overview of research concerning both acute and chronic effects of exposure to noise on children's cognitive performance. Experimental studies addressing the impact of acute exposure showed negative effects on speech perception and listening comprehension. These effects are more pronounced in children as compared to adults. Children with language or attention disorders and second-language learners are still more impaired than age-matched controls. Noise-induced disruption was also found for non-auditory tasks, i.e., serial recall of visually presented lists and reading. The impact of chronic exposure to noise was examined in quasi-experimental studies. Indoor noise and reverberation in classroom settings were found to be associated with poorer performance of the children in verbal tasks. Regarding chronic exposure to aircraft noise, studies consistently found that high exposure is associated with lower reading performance. Even though the reported effects are usually small in magnitude, and confounding variables were not always sufficiently controlled, policy makers responsible for noise abatement should be aware of the potential impact of environmental noise on children's development.

In everyday life, cognitive tasks are often performed in the presence of task-irrelevant environmental noise. Accordingly, numerous studies on noise effects on performance have been conducted since the middle of the 20th century (for reviews see Hellbrück and Liebl, 2007; Szalma and Hancock, 2011), showing that—depending on characteristics of sounds and tasks—noise of low to moderate intensity may in fact evoke substantial impairments in performance.

Most of these studies were conducted with adults. The present review, however, will focus on studies including children. Children are especially vulnerable to harmful effects of environmental noise, as cognitive functions are less automatized and thus more prone to disruption. We will report findings concerning effects of acute noise on performance in concurrent auditory and non-auditory tasks, as well as effects of chronic noise on children's cognitive development.

## Effects of acute noise on children's performance in auditory tasks

Psychoacoustic studies have consistently shown that children's speech perception is more impaired than adults' by unfavorable listening conditions. The ability to recognize speech under conditions of noise or noise combined with reverberation improves until the teenage years (Johnson, 2000; Wightman and Kistler, 2005; Talarico et al., 2007; Neuman et al., 2010). With stationary noise makers, signal-to-noise ratios (SNRs) have to be 5–7 dB higher for young children when compared to adults in order to achieve comparable levels of identification of speech or nonspeech signals, with adult-like performance reached at about 6 years of age (Schneider et al., 1989; Fallon et al., 2000; Werner, 2007). However, with maskers that vary over time, i.e., with trial-by-trial variation of the maskers' spectral composition (Oh et al., 2001; Hall et al., 2005; Leibold and Neff, 2007) or with fluctuating maskers such as single-talker speech (Wightman and Kistler, 2005), adult-like performance is usually not reached before the age of 10 years. Furthermore, children are less able than adults to make use of spectro-temporal and spatial cues for separation of signal and noise (Wightman et al., 2003; Hall et al., 2005). These findings demonstrate that children are especially prone to *informational* masking, i.e., masking that goes beyond energetic masking predicted by filter models of the auditory periphery.

Studies identified a range of linguistic and cognitive factors to be responsible for children's difficulties with speech perception in noise: concerning the former, children are less able than adults to use stored phonological knowledge to reconstruct degraded speech input. This holds for the level of individual phonemes, as children's phoneme categories are less well specified than adults' (Hazan and Barrett, 2000), but also for the lexical level since children's phonological word representations are more holistic and less segmented into phoneme units. Therefore the probability of successfully matching incomplete speech input with stored long-term representations is reduced (Nittrouer, 1996; Metsala, 1997; Mayo et al., 2003). In addition, young children are less able than older children and adults to make use of contextual cues to reconstruct noise-masked words presented in sentential context (Elliott, 1979). Concerning attention, children's immature auditory selective attention skills contribute to their difficulties with speech-in-noise perception. Children's susceptibility to informational masking has been attributed to deficits in focusing attention on auditory channels centered on signal frequencies, while ignoring nonsignal channels (Wightman and Kistler, 2005). Behavioral and ERP measures from dichotic listening paradigms provide evidence that auditory selective attention improves throughout entire childhood (Doyle, 1973; Pearson and Lane, 1991; Coch et al., 2005; Wightman et al., 2010; Gomes et al., 2012).

Owing to the mediating role of linguistic competence and selective attention, children with language or attention disorders are still more impaired than normally developing children by noise in speech perception tasks (Geffner et al., 1996; Ziegler et al., 2005, 2009). A stronger noise effect is also evident for children tested in their second language when compared to native children (Crandell and Smaldino, 1996). Studies with adults revealed that even skilled non-native listeners, whose performance in quiet is comparable to that of native listeners, are outperformed by native listeners under conditions of noise or noise combined with reverberation (Rogers et al., 2006; for review see Lecumberri et al., 2010).

Studies reviewed so far focused on simple tasks requiring identification of isolated speech targets in noise. However, listening in everyday situations, e.g., in classrooms, goes far beyond identification of single words or syllables. Effective listening in these situations requires semantic and syntactic processing of complex oral information while developing a coherent mental model of the story meaning (Kintsch, 1988). Thus, the question arises how noise affects performance in *complex* listening tasks. Studies addressing this topic revealed noise-induced decrements in adults' memory for paired associates, sequences of unrelated words, sentences, or discourse, even with SNRs allowing perfect or near-perfect identification of the speech targets (Rabbitt, 1968; Pichora-Fuller et al., 1995; Murphy et al., 2000; Ljung et al., 2009). Only a few studies in this field included children. Klatte et al. (2010a) used a listening task requiring execution of complex oral instructions and found substantial decrements due to single-talker speech and classroom noise in elementary school children. Adults were less affected. Valente et al. (2012) reported significant impairments in discourse comprehension in 8- to 12-year-olds due to broadband noise combined with reverberation. The noise effects found in these studies could not be attributed to impaired identification. A possible explanation is that identification of degraded speech requires extra resources which are then unavailable for encoding, storage, and processing of the information (McCoy et al., 2005). In addition, age-related improvements in attentional control (e.g., Davidson et al., 2006) may contribute to children's difficulties when performing listening tasks in the presence of noise. Children are less able than adults to ignore irrelevant sounds, and thus are more susceptible to sound-induced disruption in both auditory and non-auditory tasks. We will return to this point in the following section.

To summarize, the reviewed studies document that children need more favorable listening conditions than adults for decoding and processing of oral information [but see Söderlund et al. (2007, 2010) for contrasting findings in inattentive children]. This has practical implications for the acoustical design of classrooms, since effective listening is a linchpin of school learning. The issue of classroom acoustics has thus gained much interest during the past decades. Studies simulating classroom-like conditions of noise and reverberation reported severe impairments in children's listening performance (Yacullo and Hawkins, 1987; Jamieson et al., 2004; Bradley and Sato, 2008; Klatte et al., 2010a; Neuman et al., 2010; Valente et al., 2012). But even though international and national standards concerning ambient noise levels and reverberation in classrooms were developed in the past decades, many classrooms still do not fit the needs of young listeners (Bradley and Sato, 2008; Klatte et al., 2010b).

## Effects of acute noise on children's performance in nonauditory tasks

Concerning tasks that do not involve auditory targets, studies with adults have consistently shown that especially short-term memory is sensitive to negative effects of noise. Immediate serial recall of visually presented verbal items is reliably impaired by task-irrelevant sounds (for reviews see Hughes and Jones, 2001; Beaman, 2005; Schlittmeier et al., 2012). Impairments occur with single talker speech and non-speech sounds such as tones or instrumental music, but not with continuous broadband noise or babble noise. This so-called irrelevant sound effect (ISE) occurs reliably even with low-intensity sounds, with meaningless speech (e.g., speech in a language unknown to participants), and when sound presentation is confined to a rehearsal phase after encoding of the list items. However, the ISE magnitude is determined by inherent properties of the irrelevant sound. Recall performance is specifically impaired by sounds with a changing-state characteristic, i.e., by auditory streams which consist of distinct auditory–perceptive objects that vary consecutively. For example, irrelevant sounds consisting of different syllables or tones evoke an ISE, whereas steady state sounds, e.g., continuous broadband noise or repetitions of single syllables or tones, have a minor or no effect.

Different theories have been proposed concerning the underlying mechanisms of ISE evocation. Some of these assume that irrelevant sounds have automatic access to working memory, causing specific interference with the retention of cues to serial order (Jones et al., 1995) or—in case of speech—with the retention of phonological codes (Salamé and Baddeley, 1982; Neath, 2000). Other accounts attribute the ISE to the attentional burden caused by the necessity to ignore the sounds (Elliott, 2002).

Several studies found the ISE in elementary school children (Elliott, 2002; Elliott and Cowan, 2005; Klatte et al., 2007, 2010b; Elliott and Briganti, 2012), three of which including different age groups in order to learn about the role of attention in ISE evocation by analyzing developmental change. Elliott (2002) reported a dramatic increase in the magnitude of the ISE on serial recall of visually presented digits with decreasing age. Performance drop relative to quiet was 39% in the second-graders, as opposed to 11% in the adults. The age effect was interpreted as evidence for a dominant role of attentional control in ISE evocation. In a recent study of this group (Elliott and Briganti, 2012), the age effect was replicated—albeit smaller in magnitude—but other experiments in the series yielded convincing evidence against the attentional account of the ISE. Klatte et al. (2010b) used serial recall of common nouns presented pictorially and found detrimental effects due to background speech which did not differ in magnitude between first-grade children and adults. These and other findings (Hughes et al., 2007, 2012; Röer et al., 2011) suggest that two separate mechanisms contribute to noise-induced impairments in serial recall. On the one hand, irrelevant sounds with a changing state characteristic automatically interfere with maintenance of item or order information in short-term memory. This mechanism is the dominant source of disruption in the standard ISE paradigm, and seems to be adult-like in first-graders. On the other hand, irrelevant sounds may capture attention. The impact of attention capture depends on characteristics of the sound, and on the attentional abilities of the participants. Auditory events that are salient (e.g., of personal significance, such as one's own name), unexpected (e.g., slamming of a door), or deviant from the recent auditory context (e.g., change in voice in a speech stream) have a strong potential to capture attention. Children are more susceptible to sound-induced distraction due to limited attentional control. Accordingly, in Klatte et al. (2010b), first-graders were also impaired by a mixture of nonverbal classroom sounds, whereas older children and adults were unaffected.

Outside the realm of research on ISE, studies addressed effects of moderate-intensity environmental noise on children's performance in academic tasks. Early studies in this field provided little support for noise-induced impairments (Kassinove, 1972; Johansson, 1983). More recent results are inconsistent. Dockrell and Shield (2006) analyzed effects of babble and babble mixed with traffic sounds on third-graders performance in tests assessing reading, spelling, arithmetic, and attention. For all tests, overall scores were lower with babble noise when compared to quiet. Contrary to prediction, however, reading and spelling was even better in the babble plus traffic noise condition when compared to quiet and babble, and error rates in the attention test were higher in quiet when compared to both noise conditions. These results are difficult to interpret as children were not randomly assigned to noise conditions and instead were tested in their original class settings. As only two classes were assigned to each noise condition and class membership is known to affect academic performance (e.g., Kyriakides et al., 2009), a-priori group differences in the dependent variables cannot be ruled out.

A number of studies investigated the effects of background speech and transportation noise on delayed memory for texts in teenagers. Participants read prose paragraphs under different noise conditions and were later tested for prose memory in silence. Recall performance was impaired by meaningful speech (Hygge et al., 2003; Boman, 2004; Sörqvist, 2010), but not by meaningless speech (Hygge, 2003). Concerning transportation noise, results are inconsistent. Hygge (2003) found impairments due to aircraft noise during encoding. Sörqvist (2010) used a within-subjects design and found no effect of aircraft noise, but severe impairments due to meaningful speech. Hygge et al. (2003) and Hygge (2003) found impairments due to road traffic noise while Boman (2004) did not. Ljung et al. (2009) used a direct measure of online reading comprehension and found no effect of road traffic noise and meaningful speech on 12- to 13-year olds' comprehension scores.

Thus, all except one of the studies found impairments due to meaningful speech. This is in line with studies with adults, showing that meaningful speech evokes stronger impairments than meaningless speech in school-related verbal tasks involving reading (Jones et al., 1990; Oswald et al., 2000; Bell et al., 2008) or story writing (Sörqvist et al., 2012). According to the interference-by-process-account (Marsh et al., 2009), meaningful speech automatically evokes semantic processes which compete with the semantic processes involved in the task. As transportation noise does not evoke such processes, its effect on reading found in some, but not all studies, is presumably due to a more general attention-capture process. In line with this argument, Sörqvist (2010) provided evidence that the participants' attentional abilities have a stronger impact on disruption evoked by transportation noise when compared to meaningful speech. Note, however, that category membership (e.g., transportation noise vs. speech) is not sufficient to predict whether or not a sound will evoke distraction. As outlined earlier, the potential of a sound to capture attention depends on characteristics such as salience, predictability, and deviance from the recent auditory context. Thus, in addition to its specific effects on semantic processing and serial recall, speech noise containing such features is able to act as distractor (Hughes et al., 2012). On the other hand, transportation noise lacking such features has no effect on performance (Klatte et al., 2007).

## Chronic effects of noise on children's cognitive development

In view of the harmful effects of acute noise, the question arises whether enduring exposure to environmental noise may cause persisting deficits in children's cognitive development. Research in this field focused on indoor noise at school and aircraft noise. Concerning the former, studies yielded evidence for chronic effects on children's reading and prereading skills (Maxwell and Evans, 2000; Shield and Dockrell, 2008; Klatte et al., 2010c). Concerning aircraft noise, mixed results were reported with respect to chronic effects on children's attention (Stansfeld et al., 2005; van Kempen et al., 2010; Belojevic et al., 2012) and memory (Haines et al., 2001; Matheson et al., 2010), but exposure to aircraft noise was consistently associated with lower reading performance (see for review, Clark and Sörqvist, 2012). However, some of these studies are difficult to interpret due to methodological limitations. For example, cognitive abilities were usually measured in the children's regular classrooms, but acute noise levels were not always controlled. Thus, testing was done in noisy conditions for the exposed and in quiet conditions for the non-exposed children, resulting in confound of acute and chronic exposure (e.g., Seabi et al., 2012). In addition, aircraft noise has been found to be associated with socioeconomic status (SES) which in turn is strongly related to children's reading abilities. Thus, insufficient control of SES variables in early studies may have lead to an overestimation of the noise effect (Haines et al., 2002).

The hitherto most comprehensive study in this field, the cross-sectional RANCH (road-traffic and aircraft noise exposure and children's cognition and health) study (Stansfeld et al., 2005) included children (*N* = 2844) living in the vicinity of huge international airports in the UK, the Netherlands, and Spain. Whereas prior studies confined to comparisons of highly exposed and non- exposed children, noise exposure in the RANCH study was included as continuous variable, aiming to reveal the noise levels at which the harmful effects on children's cognition begin. With SES being controlled, the authors found no effect of aircraft noise exposure on sustained attention, working memory, and delayed recall of orally presented stories, but a linear exposure–effect relationship between aircraft noise and decreasing reading comprehension. This effect is often cited as evidence for a causal role of aircraft noise in reading impairment. What is often unreported in the secondary literature is, however, that there was another exposure–effect relationship, revealing *enhanced* performance in episodic memory with increasing exposure to road traffic noise. This counter-intuitive finding remains unexplained.

Concerning the underlying mechanisms of chronic noise effects, some authors proposed that enduring exposure to noise in early childhood affects the development of basic language functions which are of special importance in reading acquisition (Evans and Maxwell, 1997; Maxwell and Evans, 2000; Klatte et al., 2010c). This is a reasonable argument in view of, on the one hand, the vulnerability of children's speech perception and short-term memory for disruption due to acute noise, and on the other hand, the important role of these functions in reading acquisition (Baddeley et al., 1998; Steinbrink and Klatte, 2008; Ziegler et al., 2009). In line with this argument, electrophysiological studies revealed alterations in the cortical responses to speech sounds in individuals with a long-term exposure to occupational noise (Brattico et al., 2005).

## Conclusions

The reviewed studies document harmful effects of noise on children's learning. Children are much more impaired than adults by noise in tasks involving speech perception and listening comprehension. Non-auditory tasks such as short-term memory, reading and writing are also impaired by noise. Depending on the nature of the tasks and sounds, these impairments may result from specific interference with perceptual and cognitive processes involved in the focal task, and/or from a more general attention capture process.

Concerning chronic effects, despite inconsistencies within and across studies, the available evidence indicates that enduring exposure to environmental noise may affect children's cognitive development. Even though the reported effects are usually small in magnitude, they have to be taken seriously in view of possible long-term effects and the accumulation of risk factors in noise-exposed children (Evans, 2004). Obviously, the findings reported in this review have practical implications for the acoustical design of schools, for the placement of schools in the vicinity of airports, and for the policy of noise abatement.

### Conflict of interest statement

The authors declare that the research was conducted in the absence of any commercial or financial relationships that could be construed as a potential conflict of interest.
